# Electronic Health Literacy Among Magnetic Resonance Imaging and Computed Tomography Medical Imaging Outpatients: Cluster Analysis

**DOI:** 10.2196/13423

**Published:** 2019-08-28

**Authors:** Lisa Lynne Hyde, Allison W Boyes, Lisa J Mackenzie, Lucy Leigh, Christopher Oldmeadow, Carlos Riveros, Rob Sanson-Fisher

**Affiliations:** 1 School of Medicine and Public Health Faculty of Health and Medicine University of Newcastle Callaghan Australia; 2 Priority Research Centre for Health Behaviour University of Newcastle Callaghan Australia; 3 Hunter Medical Research Institute New Lambton Heights Australia; 4 Hunter Cancer Research Alliance Newcastle Australia

**Keywords:** internet, health, literacy, cluster analysis, medical imaging

## Abstract

**Background:**

Variations in an individual’s electronic health (eHealth) literacy may influence the degree to which health consumers can benefit from eHealth. The eHealth Literacy Scale (eHEALS) is a common measure of eHealth literacy. However, the lack of guidelines for the standardized interpretation of eHEALS scores limits its research and clinical utility. Cut points are often arbitrarily applied at the eHEALS item or global level, which assumes a dichotomy of high and low eHealth literacy. This approach disregards scale constructs and results in inaccurate and inconsistent conclusions. Cluster analysis is an exploratory technique, which can be used to overcome these issues, by identifying classes of patients reporting similar eHealth literacy without imposing data cut points.

**Objective:**

The aim of this cross-sectional study was to identify classes of patients reporting similar eHealth literacy and assess characteristics associated with class membership.

**Methods:**

Medical imaging outpatients were recruited consecutively in the waiting room of one major public hospital in New South Wales, Australia. Participants completed a self-report questionnaire assessing their sociodemographic characteristics and eHealth literacy, using the eHEALS. Latent class analysis was used to explore eHealth literacy clusters identified by a distance-based cluster analysis, and to identify characteristics associated with class membership.

**Results:**

Of the 268 eligible and consenting participants, 256 (95.5%) completed the eHEALS. Consistent with distance-based findings, 4 latent classes were identified, which were labeled as low (21.1%, 54/256), moderate (26.2%, 67/256), high (32.8%, 84/256), and very high (19.9%, 51/256) eHealth literacy. Compared with the low class, participants who preferred to receive a lot of health information reported significantly higher odds of moderate eHealth literacy (odds ratio 16.67, 95% CI 1.67-100.00; *P*=.02), and those who used the internet at least daily reported significantly higher odds of high eHealth literacy (odds ratio 4.76, 95% CI 1.59-14.29; *P*=.007).

**Conclusions:**

The identification of multiple classes of eHealth literacy, using both distance-based and latent class analyses, highlights the limitations of using the eHEALS global score as a dichotomous measurement tool. The findings suggest that eHealth literacy support needs vary in this population. The identification of low and moderate eHealth literacy classes indicate that the design of eHealth resources should be tailored to patients’ varying levels of eHealth literacy. eHealth literacy improvement interventions are needed, and these should be targeted based on individuals’ internet use frequency and health information amount preferences.

## Introduction

### Electronic Health Literacy Is Important for the Use and Receipt of Benefits From Electronic Health Programs

Web-based interventions have been reported to be consistently more effective than non-Web-based modalities in changing patient health behaviors and health-related knowledge [1]. Information and communication technology is also recognized as a promising enabler of safe, integrated, and high-quality health care, yet more scientifically rigorous research is needed [2,3]. Accordingly, internet-enabled health care is a strategic priority globally [4-7]. Electronic health (eHealth) literacy is one important factor influencing the use and receipt of benefits from Web-based health resources [8-10]. eHealth literacy refers to an individual’s ability to seek, find, understand, and appraise health information from electronic sources, and apply the knowledge gained to addressing or solving a health problem [11]. The concept is derived from 6 literacy types (ie, health, computer, media, science, information, traditional literacy, and numeracy), which play an important role in facilitating engagement with Web-based health resources [11]. Inadequate eHealth literacy has been self-reported as a barrier to use of the internet for health information seeking purposes among the chronically ill [12]. Furthermore, descriptive research indicates that eHealth literacy is associated with positive cognitive (eg, understanding of health status) [8], instrumental (eg, self-management, physical exercise, and dieting) [8-10], and interpersonal (eg, physician interaction) [8] outcomes from Web-based health information searches. Individuals with lower eHealth literacy have been suggested to be older [8,13,14], less educated [8,14,15], have lower access to, or use of, the internet [15-17], and have poorer health [8].

### Interpretations of Electronic Health Literacy Data are Inconsistent

Approaches used to assess eHealth literacy have included objective performance testing [18,19] and self-reported measurement [20-23]. The most commonly used self-reported measure is the 8-item, eHealth Literacy Scale (eHEALS) [20]. Compared with other self-report measures of eHealth literacy, strengths of the eHEALS include its psychometric rigor, brevity, ease of administration, and availability in a number of languages [17,19,20,24-26]. One of the key issues limiting the utility of the eHEALS is the lack of information about interpretation of these data. Although there is a convention that higher scores represent a higher level of eHealth literacy [20], there is an absence of guidance for the standardized interpretation of these scores. This guidance is needed to inform decision-making and follow-up actions [27]. eHEALS mean and median scores [8,13,14,28], as well as item response frequencies [14,29,30], are typically reported. Cut points have been arbitrarily applied at the item level [15], which disregards scale constructs. Furthermore, the common use of a single cut point to the global scale [8,16,28] implies a dichotomy of high versus low eHealth literacy and does not account for respondent self-perceived competency across the multiple eHEALS factors (ie, awareness, skills, and evaluation) [24,31]. These factors have only recently been identified [24,31], demonstrating that our understanding of the eHEALS and its psychometric properties is continuing to evolve more than a decade after the scale was published.

### A Robust Approach to Analyzing Electronic Health Literacy Data Is Required

Shortcomings in the interpretation of eHEALS scores highlight the need for a robust approach to analyzing and interpreting eHealth literacy data. In line with the principles of scale development [27,32], measures should be refined as new data about a scale’s properties accumulates. This includes retesting a scale when it is used in new populations and as new analytical techniques become available [27,32]. Cluster analysis is a sophisticated analytical approach, which has not previously been applied to eHealth literacy research. This powerful technique is used to identify natural groupings or structures within data and can therefore classify individuals who score similarly on an outcome measure, such as the eHEALS [33]. It has several strengths including: First, it is a data-driven exploratory technique and therefore not dependent on scoring thresholds, which are arbitrarily imposed by the author(s). Second, being able to observe and characterize natural structures or groupings means that researchers have a better understanding of subgroups of eHealth literacy in the sample population. If classes (or clusters) exist, ignoring their presence by analyzing the data as a single group could lead to an averaging out of any effects of interest [34]. Third, this approach allows for the multiple eHEALS domains (ie, skill, awareness, and evaluate) to be considered simultaneously across subgroups. For example, it can be known if one subgroup self-rates their *awareness* as highest, whereas another subgroup self-rates their *skills* as highest. Finally, regression analyses can be completed to examine patient characteristics associated with assignment to each eHealth literacy class.

By understanding the number and characteristics of groupings, it can be known whether a one size fits all approach to eHealth literacy improvement is appropriate, or whether more tailored interventions are required. If tailoring is needed, understanding how different classes scored across the eHEALS factors allows researchers and clinicians to ensure interventions are designed to specifically address the needs of that subgroup. Furthermore, understanding patient characteristics associated with class membership allows the identification of individuals who should be targeted for interventions, or who will require more intensive support throughout periods of eHealth delivery. A cluster analysis of eHEALS data is therefore an important next step to better understand the multicomponent nature of eHealth literacy and how these eHEALS factors coexist in subgroups of patients.

This study aimed to determine (1) whether there are identifiable eHealth literacy classes among magnetic resonance imaging (MRI) and computed tomography (CT) medical imaging outpatients; and (2) sociodemographic and internet use characteristics associated with each eHealth literacy class.

## Methods

### Design and Setting

This cross-sectional study was completed with MRI and CT medical imaging outpatients attending the imaging department of a large, tertiary hospital, located within New South Wales, Australia. The results of this study have been reported in accordance with the Strengthening the Reporting of Observational Studies in Epidemiology checklist [35] and the Checklist for Reporting Results of Internet E-Surveys [36].

### Participants

Eligible participants were: (1) attending for an outpatient MRI or CT scan; (2) 18 years or older; and (3) reported having access to the internet for personal use. Participants were excluded if they were: (1) non-English speaking; (2) deemed by reception staff to be cognitively or physically unable to consent or complete the survey; or (3) identified as having completed the survey previously. MRI and CT medical imaging outpatients were the focus of this research because they have high unmet information preferences, which could potentially be met by eHealth capabilities [37].

### Procedure

Medical imaging department receptionists identified potentially eligible participants when they presented for their outpatient appointment. Potentially eligible participants were informed about the research and invited to speak with a trained research assistant. Interested patients were provided with a written information sheet and introduced to the research assistant, who gave an overview of the study and obtained the patient’s verbal consent to participate. During this overview, interested patients were told that the Web-based questionnaire would take approximately 10-15 mins to complete, participation was voluntary, and responses would remain confidential. The age, gender, and scan type of noninterested and nonconsenting patients were recorded. Consenting patients were provided with a tablet computer and asked to complete a Web-based questionnaire before their scan. Participants’ study identification number, assigned by the receptionist and entered by the research assistant, provided access to the questionnaire. Each participant could move freely through each screen using next and back buttons. The questionnaire was pilot tested with MRI and CT medical imaging outpatients 2 weeks before study commencement, which confirmed the acceptability and feasibility of electronic survey administration in this study setting. A paper-and-pen version of the questionnaire was available to participants who requested it. If the patient was called for their procedure before finishing the questionnaire, only those questions that had been completed were used for data analysis. Electronic responses were deidentified, collected using the QuON platform [38], and stored securely on an access-restricted part of the University of Newcastle server. Ethics approval was obtained from the Human Research Ethics Committees of the Hunter New England Local Health District (16/10/19/5.11) and University of Newcastle (H-2016-0386).

### Measure

*eHealth literacy* was assessed using the 8-item eHEALS. All 8 eHEALS items were administered on 1 screen within the Web-based questionnaire, and the presentation of these items was not random. Respondents indicated their level of agreement with each statement on a 5-point Likert scale from 1 *strongly disagree* to 5 *strongly agree*. Responses were summed to give a final score ranging from 8 to 40, with higher scores indicating higher eHealth literacy. The tool has demonstrated test-retest reliability [17], internal consistency [17,19,28], and measurement invariance across English speaking countries [24]. Previous studies, largely employing exploratory factor analysis, have suggested that the scale measures a single factor [8,17,19,20]. Emerging research using confirmatory factor analysis and based on the theoretical underpinnings of eHealth literacy suggests that the scale measures 3 factors: awareness, skills, and evaluate [24,31]. This 3-factor eHEALS structure has been identified in the medical imaging study setting (standardized root mean residual=0.038; confirmatory fit index=0.944; and root mean square error of approximation=0.156) [31]. As such, self-rated awareness, skills, and evaluate competencies of patients within each subgroup were explored within this study.

### Study Factors

On the basis of previous research indicating an association with eHealth literacy, standard self-report items assessed participant gender, age, marital status, education, internet use frequency, and overall health status [8,13-17]. Remoteness of residence, health information amount preference (no information; some information; and a lot of information), and internet use for scan preparation (yes; no; and don’t know) were hypothesized to influence eHealth literacy and were, therefore, included as covariates. Participant postcodes were mapped to the Accessibility/Remoteness Index of Australia Plus to categorize participant remoteness as metropolitan (major cities of Australia) or nonmetropolitan (inner regional, outer regional, remote, or very remote Australia) [39].

### Data Analysis

Participant characteristics were summarized as frequencies and percentages or means and standard deviations. Consent bias was assessed for gender, scan type, and age group using Chi-square tests. Given the high completion rate (98.1%, 256/261 for individuals starting eHEALS items), only complete eHEALS data were included in the analyses. Items relating to each eHEALS factor were summed to generate separate awareness, skill, and evaluate factor scores.

#### Identification of Electronic Health Literacy Classes

Cluster analysis was completed using a 2-phased approach. Distance-based unsupervised clustering was undertaken as an initial exploratory knowledge discovery technique, to identify natural clusters of patients according to their responses (refer Multimedia Appendix 1 for methods and results). Secondary clustering of patients, using latent class analysis (LCA) as a statistical modeling approach, was to be completed as a follow-up if distance-based cluster structures were observed. LCA was subsequently performed to verify the 4-cluster structure identified. LCA is less sensitive to choice of parameters (eg, distance metric), allows for uncertainty in class membership, and has greater power and lower type 1 error rates when compared with other clustering techniques [34], and was, therefore, selected as the primary analysis technique. Latent class membership probabilities were calculated to determine the proportion of the sample that belonged to each of the classes. Item response probabilities were calculated to determine the probability of endorsing each response option, conditional on class membership. The Bayesian Information Criterion (BIC) and G^2^-statistic were computed to aid in determining the optimal number of classes (with plateauing indicating no improvements to model fit) [40], as were overall class interpretability and model parsimony. Model entropy was computed, with values closer to 1 representing clear class delineation [41]. The maximum posterior probability of class membership was also calculated for each participant, based on the optimal number of classes, with values greater than .5 indicating adequate probability for class assignment [42].

#### Characteristics Associated With Class Membership

An LCA regression analysis was performed to identify participant sociodemographic and internet use characteristics associated with class membership. Given the exploratory nature of data analysis, all covariates were initially cross-tabulated with class membership (assigned according to maximum posterior probability) to identify model sparseness, and then analyzed using univariate LCA regression: gender; age (<65 years vs 65+ years); geographic location of residence (major city vs regional or rural); marital status (married or living with partner vs not married); education (high school or less vs more than high school); overall health (fair or worse; good or better than good); information amount preference (a lot of information vs not a lot of information); internet use for scan preparation; and internet use frequency (daily vs less than daily). Likelihood ratio tests (based on the univariate results) were performed to determine whether each predictor significantly improved the fit of the model. Covariates with a statistically significant likelihood ratio test (*P*<.05) were included in the final multivariable LCA regression. Distance-based and latent class analyses were performed in R 3.4 [43]. Descriptive statistics were computed in STATA v13.

### Sample Size

Sample sizes of at least 200 have been suggested as adequate for LCA, dependent on subsequent model fit and number of classes [40,44]. As such, a sample of at least 200 was deemed appropriate for this study.

## Results

### Sample

A total of 405 potentially eligible patients were invited to discuss the study with a research assistant during the 7-week recruitment period, of which 354 (87.4%) were interested in participating. Of 268 eligible participants, 261 (97.4%) started the eHEALS, 256 (95.5%) completed all eHEALS items, and 222 (82.8%) completed all eHEALS and study factor items. There were no significant differences between patients who were and were not interested in participating in the study based on gender, scan type, or age group. Table 1 provides a summary of the sociodemographic, scan, and internet characteristics of the study sample.

**Table 1:** Participant sociodemographic, scan, and internet characteristics (N=256). Number of observations for each characteristic may not total 256 because of missing data.

Characteristic		Value
Age (years), mean (SD)		53 (15.0)
**Electronic Health Scale (eHEALS) domain score, mean (SD)**		
	Awareness (possible total=10)	6.9 (2.0)
	Skills (possible total=15)	10.9 (2.9)
	Evaluate (possible total=15)	10.0 (3.1)
**Gender, n (%)**		
	Male	112 (43.8)
	Female	144 (56.3)
**Marital status, n (%)**		
	Married or living with partner	146 (64.6)
	Not married or living with partner	80 (35.4)
**Education completed, n (%)**		
	High school or less	128 (56.6)
	More than high school	98 (43.4)
**Geographic location, n (%)**		
	Metropolitan	200 (78.1)
	Nonmetropolitan	56 (21.9)
**Overall health, n (%)**		
	Poor	17 (7.7)
	Fair	75 (34.1)
	Good	94 (42.7)
	Very good	34 (15.5)
**Scan type, n (%)**		
	Computed tomography	101 (39.4)
	Magnetic resonance imaging	152 (59.4)
	Don’t know	3 (1.2)
**Used internet for scan, n (%)**		
	Yes	27 (10.5)
	No	228 (89.1)
	Don’t know	1 (0.4)
**Frequency of internet use, n (%)**		
	Less than once a month	11 (4.3)
	Once a month	5 (1.9)
	A few times a month	14 (5.5)
	A few times a week	33 (12.9)
	About once a day	47 (18.4)
	Several times a day	146 (57.0)
**Information amount preference, n (%)**		
	No information	2 (0.8)
	Some information	58 (25.9)
	A lot of information	165 (73.3)

### Identification of Electronic Health Literacy Classes

The BIC and G^2^-statistic continued to decrease as the number of classes (K) increased, but the improvement was progressively smaller after 3 classes (see Table 2). On the basis of the interpretability of the latent classes, the reduction in class size beyond K=4, and parsimony, the 4 class model was selected as the optimal class structure. The lowest maximum posterior probability under this 4 class model was .516. As such, all participants exceeded the threshold of .5 for maximum posterior probability and were assigned to a class. Hence, LCA findings on number of classes were consistent with that of distance-based clustering (see Multimedia Appendix 1).

Multimedia Appendix 2 shows the unconditional item response probabilities of each eHEALS response option based on class assignment. Classes were named according to likely level of eHealth literacy, with respect to that of other classes identified in the analysis:

- Class 1—*low eHealth literacy* (21.1% of respondents, 54/256): when compared with other classes, class 1 had the highest probability of responding *disagree* and *strongly disagree* across all eHEALS items. The probability of this group responding either *disagree* or *strongly disagree* was highest for awareness items (0.88 and 0.89), followed by evaluate items (0.79, 0.81, and 0.88) and skills items (0.66, 0.75, and 0.84).
- Class 2—*moderate eHealth literacy* (26.2% of respondents, 67/256): when compared with other classes, class 2 had the highest probability of responding *undecided* across all eHEALS items, and the second highest probability of responding *agree* across awareness and skills items. This group was most likely to respond *undecided* to awareness items (0.56 and 0.59), either agree (0.54 and 0.58) or undecided (0.48) to skills items, and undecided to evaluate items (0.55, 0.61, and 0.63).
- Class 3—*high eHealth literacy* (32.8% of respondents, 84/256): when compared with other classes, class 3 had the highest probability of responding *agree* across all eHEALS items. The probability of this class responding *agree* was greatest for skills items (0.97, 0.97, and 1.00), followed by awareness (0.80 and 0.91), and evaluate items (0.68, 0.71, and 0.81).
- Class 4—*very high eHealth literacy* (19.9% of respondents, 51/256): when compared with other classes, class 4 had the highest probability of responding *strongly agree* across all eHEALS items. The probability of this class responding *strongly agree* was greatest for skills items (0.71, 0.79 and 0.90), followed by evaluate (0.57, 0.74, and 0.86), and awareness items (0.53 and 0.61).

### Characteristics Associated With Class Membership

Internet use for scan preparation was not included in regression analyses because of sparseness (ie, 10.5%, 27/256 of participants responded *yes* to internet use for scan preparation). Following univariate analyses, likelihood ratio difference tests indicated that age; education, marital status, overall health status, information amount preference, and internet use frequency all significantly improved the fit of the model (*P*<.05; see Multimedia Appendix 3) and were included in the multivariable regression analysis (see Table 3).

Class 1 (low eHealth literacy) was selected as a reference class for multivariable regression. This was because these participants likely need additional support to engage with eHealth, making identification of the characteristics of participants in this subgroup a priority. As shown in Table 3, participants who indicated that they preferred not to receive a lot of information about their health had 0.06 times the odds of belonging to class 2 (moderate eHealth literacy), compared with class 1 (low eHealth literacy), and this difference was statistically significant. Furthermore, participants who reported using the internet less than daily had 0.21 times the odds of belonging to class 3 (high eHealth literacy), compared with class 1 (low eHealth literacy), and this difference was statistically significant. There were no other significant differences in sociodemographic or internet use attributes between participants in class 1 (low eHealth literacy) and classes 2, 3, and 4 (moderate, high, and very high eHealth literacy, respectively).

**Table 2:** Goodness of fit indices for 1 to 5 class structures.

Class structure	BIC^a^	G^2^-statistic	Entropy
1 class structure	5893.74	3402.83	1.00
2 class structure	5148.66	2474.76	0.97
3 class structure	4651.68	1794.79	0.98
4 class structure	4556.81	1516.93	0.92
5 class structure	4545.21	1322.34	0.90

^a^BIC: Bayesian Information Criterion.

**Table 3:** Adjusted odds ratios associated with membership of classes 2, 3, and 4, compared with class 1.

Variable		Class 1 versus class 2 (low vs moderate)		Class 1 versus class 3 (low vs high)		Class 1 versus class 4 (low vs very high)	
		Odds ratio (95% CI)	*P* value	Odds ratio (95% CI)	*P* value	Odds ratio (95% CI)	*P* value
**Age**							
	<65 years	Ref^a^	Ref	Ref	Ref	Ref	Ref
	65 years or older	0.37 (0.06-2.11)	.26	0.32 (0.10-1.03)	.06	0.37 (0.07-2.00)	.25
**Education**							
	High school or less	Ref	Ref	Ref	Ref	Ref	Ref
	More than high school	1.09 (0.15-7.65)	.93	2.21 (0.52-9.47)	.29	3.89 (0.67-22.76)	.14
**Marital status**							
	Married or living with partner	Ref	Ref	Ref	Ref	Ref	Ref
	Not married or living with partner	1.63 (0.26-10.23)	.60	0.96 (0.27-3.41)	.96	0.91 (0.14-6.01)	.92
**Information amount preference**							
	A lot of information	Ref	Ref	Ref	Ref	Ref	Ref
	Not a lot of information	0.06 (0.01-0.60)	.02^b^	0.61 (0.18-2.04)	.43	0.23 (0.04-1.29)	.10
**Overall health**							
	Fair or worse	Ref	Ref	Ref	Ref	Ref	Ref
	Good or better than good	1.10 (0.24-5.02)	.91	1.16 (0.35-3.87)	.81	1.48 (0.33-6.68)	.61
**Internet use frequency**							
	Daily	Ref	Ref	Ref	Ref	Ref	Ref
	Less than once a day	0.62 (0.14-2.67)	.52	0.21 (0.07-0.63)	.007^b^	0.17 (0.02-1.76)	.14

^a^Ref: reference category.

^b^Statistically significant.

## Discussion

### Principal Findings

This study was the first to identify classes of patients based on eHealth literacy, and to assess characteristics associated with class membership. The identification of multiple classes, using both distance-based and latent class analyses, highlights issues with using the eHEALS global score as a dichotomous measurement tool. In particular, these findings suggest that it may be important to account for multiple eHealth literacy subgroups when developing standardized guidance for the interpretation of eHEALS scores. Furthermore, the identification of multiple classes suggests that the design and delivery of eHealth resources may need to be tailored based on eHealth literacy. Patient characteristics, such as internet use frequency and health-related information amount preferences, may provide an indication of eHealth literacy, and related support needs.

### Multiple Electronic Health Literacy Subgroups Were Identified

In total, 4 eHealth literacy classes were identified, and the probabilities of belonging to each of the 4 classes were similar (ie, range 19.9%-32.8%). The finding that eHealth literacy varied substantially in this population suggests that MRI and CT medical imaging outpatients may have differing support needs relating to the use of eHealth technology. Subgroups of patients were characterized by having either very high, high, moderate, or low eHealth literacy. Within the very high eHealth literacy subgroup, awareness was the lowest scoring competency. This may be because consumers who are familiar with eHealth also understand the masses of Web-based information that is available and the common difficulty of locating valid and reliable information sources [12]. Across all classes, participants reported being most competent in their skills using eHealth resources. Such skills may be perceived highly because they align to the computer and media literacy types, which comprise eHealth literacy [11]. These literacy types are increasingly used in the digital era, with 87% of Australians being identified as internet users in 2016-2017 [45].

In total, 2 out of 4 classes, comprising 52.7% of respondents, had the highest probability of responding either agree or strongly agree to eHEALS items, reflecting high and very high eHealth literacy. Despite this, there was room for improvement in awareness, skills, and evaluation competencies for the remaining 2 classes, comprising 47.3% of respondents and reflecting low and moderate eHealth literacy. This approximately even split in eHealth literacy capabilities is also apparent in other studies completed with cardiovascular disease patients [16] and chronic disease patients [46], which used arbitrary cut points to dichotomize high versus low eHealth literacy. It is possible that the application of dichotomous cut points prevented the identification of such diverse eHealth literacy subgroups. Further research using cluster analyses should be conducted to determine whether multiple eHealth literacy subgroups exist across other health consumer populations. This information may inform the development of more targeted eHealth literacy improvement interventions.

### Internet Use Frequency and Health Information Amount Preferences Predicted Class Membership

Those who had used the internet less than daily had approximately 5 times the odds of belonging to the low eHealth literacy class compared with the high eHealth literacy class. Although mixed findings exist [19], an association between internet use and eHealth literacy has been reported in studies with chronically ill patients and the general public [15-17]. Our findings may suggest that frequent internet users do use the internet for health, and this may result in greater self-reported eHealth literacy. Alternatively, they may indicate that frequent internet users self-perceive that their ability to engage with and evaluate general internet resources is transferable to health-related content.

Those with a preference not to receive a lot of information about their health had over 16 times the odds of belonging to the low eHealth literacy class, compared with the moderate eHealth literacy class. To the authors’ knowledge, this study is the first to explore the association between preferred amount of information and eHealth literacy. It is possible that the inclusion of an *undecided* response option resulted in imposter syndrome for those in the moderate class [47]. In this case, participants underestimate their competency, opting for a neutral response option, to prevent being perceived as overconfident. Therefore, those in the moderate class may be more eHealth literate than findings suggest, which could contribute to a significant finding when comparing low and moderate classes. It may also be possible that those who prefer to receive a lot of information about their health are Web-based health-related information seekers, hence requiring eHealth literacy. An evidence review completed by the Australian Commission on Quality and Safety in Health Care found that patients typically use the internet as a supplement to advice from a health professional [48]. It is therefore likely that those who have greater preferences for health-related information require and develop the awareness, skills, and evaluation abilities needed to use this Web-based supplementary information. An analysis of the potentially moderating effects of Web-based health-related information seeking on the association between information amount preference and eHealth literacy should be explored in the future. This analysis should include an examination of the types of eHealth resources being accessed and used.

The technology acceptance model provides a theoretical justification for the characteristics related to a subgroup assignment [49]. Under this model, technology acceptance is influenced by perceived ease of use, and usefulness of the internet [49]. Accordingly, those who use the internet more frequently may be more likely to perceive ease of use of Web-based health resources. Similarly, those who prefer to receive a lot of health-related information may be more likely to deem eHealth as useful. Such perceived acceptability may result in greater self-rated eHealth literacy. Continued studies are needed to investigate this association and determine whether other factors not explored in this study, which promote perceived ease of use and usefulness of eHealth (eg, speed and availability of the internet, and self-management of chronic conditions, respectively), are associated with eHealth literacy. Contrary to expectations and inconsistent with previous studies [8,13-15], no other examined sociodemographic characteristics significantly influenced class membership. Inconsistencies with existing literature may indicate that the predictors of eHealth literacy differ across populations, settings, or cut points applied.

### Practice Implications

The identification of low and moderate eHealth literacy classes suggests that eHealth literacy improvement interventions may be warranted within this population. However, there is minimal high-quality research investigating the effectiveness of such interventions, highlighting a need for continued research in this area [50]. Given their association with low class membership, those who use the internet less than daily and prefer not to receive a lot of health information should be the focus of such eHealth literacy improvement interventions. In the interim, researchers and clinicians should tailor the design and delivery of eHealth resources to patients’ eHealth literacy, to maximize engagement and potential receipt of benefits. As skills were the highest rated competency across all classes within this study population, future eHealth interventions should be designed with a focus on promoting awareness and reducing the need to evaluate eHealth resources within the imaging setting. A written provider recommendation, which directs consumers toward credible eHealth resources, may be one scalable strategy to do this [31,51]. In cases where skills are low, alternative strategies may be needed, such as clear instructions on how to appropriately navigate Web-based content, reduced click-through requirements to retrieve Web-based materials, and the use of persuasive system design elements to enhance usability and maintain engagement [52].

### Limitations and Future Research

To aid in the interpretation of findings, labels (ie, very high, high, moderate, and low) were arbitrarily assigned to eHealth literacy classes. It is therefore unclear whether, for example, those classified as very high eHealth literacy were indeed very high. As this study applied a novel approach to data analysis and interpretation, the generalizability of findings across medical imaging settings and to other patient groups is unknown. This class structure and the predictors of class membership should be studied and replicated in other populations. Furthermore, it is possible that the setting influenced responses as participants may have assumed that eHEALS questions related to scan-specific information on the internet rather than general eHealth resources.

The eHEALS was selected because of its established psychometric properties, emerging research proposing a 3-factor structure, and wide application [17,19,20,24,28,31]. However, it has been criticized for not measuring health 2.0. (ie, user-generated content and interactivity) and, therefore, lacking relevance to modern technology [21,24,53]. Some studies have adapted the scale to address this limitation, yet the body of research is small and as a result, the impacts on scale psychometric properties remain unclear [21,24]. The generation of new Web-based content is, however, not highly relevant within the context of preparatory information provision for medical imaging procedures and this limitation is, therefore, not expected to influence our study.

### Conclusions

This study used sophisticated analytical techniques to add to evidence about the nature of eHEALS scores within a clinical population. Cluster analyses were used to identify 4 classes of patients with differing eHealth literacy within this sample of MRI and CT medical imaging outpatients. The proportion of participants assigned to each latent class was similar, suggesting that eHealth literacy varies within this study setting. Across all classes, skills were perceived as the highest rated competency followed by either awareness or evaluation. The frequency of participants’ personal internet use and their health-related information preferences predicted class membership. Tools such as the eHEALS may need to be administered to identify class assignment, and inform eHealth literacy improvement interventions, as well as the design and delivery of eHealth resources. Findings from this study should also contribute to the development of guidance for eHEALS scoring interpretation, which is a necessary next step to improve scale utility [27]. Study findings should be replicated in other populations and settings to increase the generalizability of results.

## Appendix

Multimedia Appendix 1 Distance-based cluster analysis.

Multimedia Appendix 2 Unconditional item response probabilities for a 4-class model of electronic health literacy.

Multimedia Appendix 3 Log likelihood difference tests.

## Abbreviations

BIC: Bayesian Information Criterion
CT: computed tomography
eHEALS: eHealth Literacy Scale
eHealth: electronic health
LCA: latent class analysis
MRI: magnetic resonance imaging
