# Supplementary material for: Electronic Health Literacy Among Magnetic Resonance Imaging and Computed Tomography Medical Imaging Outpatients: Cluster Analysis
Source: J Med Internet Res. 2019 Aug 28;21(8):e13423. doi: 10.2196/13423 (PMC6737886; doi:10.2196/13423)
Supplement: Multimedia Appendix 1 [file jmir_v21i8e13423_app1.pdf]

Multimedia Appendix 1: Distance-based cluster analysis

Method

Distance-based cluster analysis was used as an unsupervised, exploratory, knowledge discovery technique to identify natural clusters of participants reporting similar eHealth literacy. Euclidean distance (i.e. straight line distance between coordinates) and Cosine distance (angular distance between coordinates) was computed. Hierarchical and kmeans clustering was computed on the optimal distance metric [1].

Hierarchical clustering is an approach in which points with the closest distance are gradually combined in a tree structure, and (in the simplest case) a fixed-depth cut point is selected, hence establishing clusters following the branches of the tree [1]. Kmeans clustering starts from a number of *k* samples randomly chosen, and iteratively adds samples to one of each *k* groups by choosing the group with the closest mean to the sample. After a sample is added to a group, the group mean position is updated. The procedure is repeated several times with different random starting points to robustly estimate group assignment [1].

The gap statistic, which uses bootstrapping to compare within cluster dispersion for a varying number of clusters to that of a reference uniform distribution, was computed for kmeans and hierarchical clustering. The point at which the gap statistic was greatest, was taken to indicate the optimal number of clusters. Clustering structure was visually appraised by a t-distribution stochastic network embedding (TSNE) projection [2, 3], with clusters indicated in different colours.

Results

Euclidean distance provided an optimal distance metric

Plots indicated a more compact and differentiated model using Euclidean distance (refer Suppl. Figure 1), with the overall dispersion of the points narrower when compared to that of the Cosine distance plot (refer Suppl. Figure 2). In both cases, distinctive cluster structures were visually apparent. This may be because the eHEALS response scale is ordinal (i.e. participants had only 5 response options), and distance between samples can only take discrete rather than continuous values.

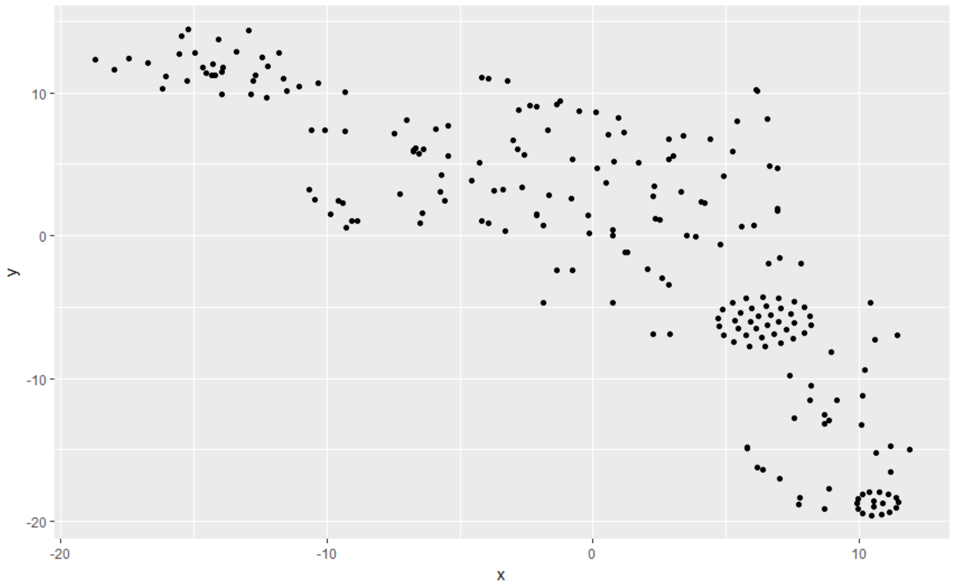

Suppl. Figure 1: Euclidean TSNE Projection

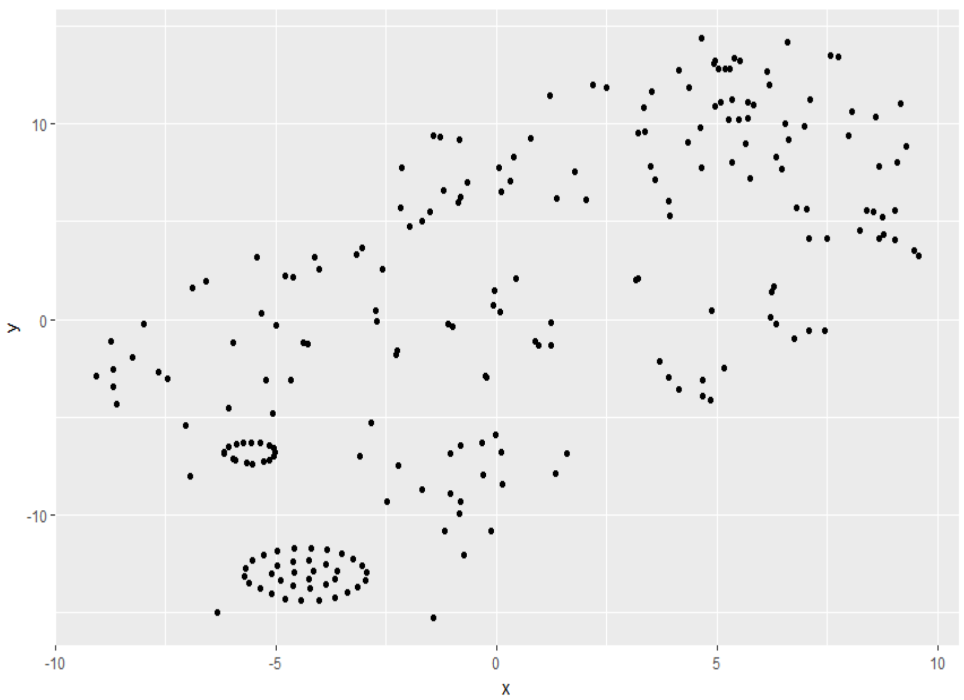

Suppl. Figure 2: Cosine TSNE Projection

The gap statistic for hierarchical clustering indicated a four-cluster structure

The gap statistic computed for hierarchical clustering of the Euclidean distance metric indicated a four cluster structure (refer Suppl. Figure 3): cluster 1: n = 135; cluster 2: n = 9; cluster 3: n = 44; cluster 4: n = 68. This cluster structure was apparent based on both firstmax (i.e. first maximum of the gap statistic) and globalmax (i.e. highest gap statistic in the range analysed) criteria. The hierarchical clustering dendrogram illustrates this four-cluster structure (refer Suppl. Figure 4), with high branch joining points demonstrating clear delineation between clusters. This four cluster structure was also apparent when plotted against TSNE projections (refer Suppl. Figure 5). The gap statistic computed for kmeans clustering did not indicate an optimal number of clusters (refer Suppl. Figure 6).

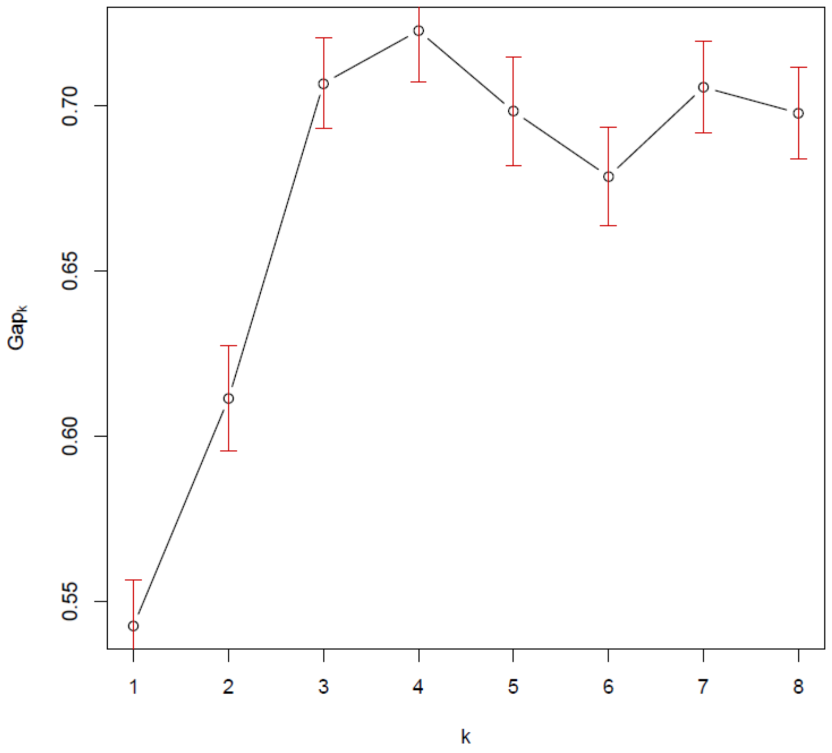

Suppl. Figure 3: Gap statistic computed for hierarchical clustering of eHEALS items

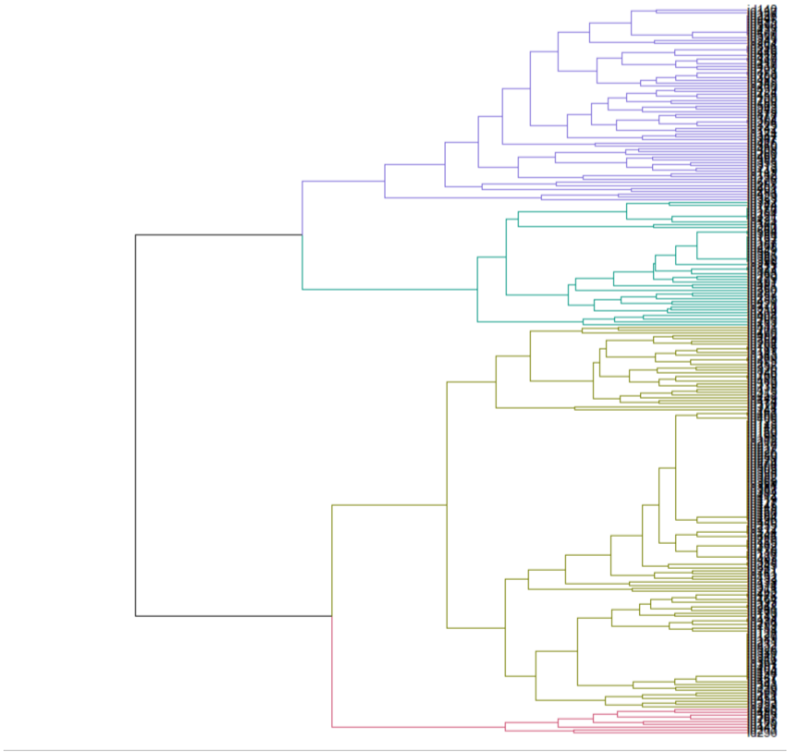

Suppl. Figure 4: Hierarchical clustering dendrogram (K=4) on Euclidean distance

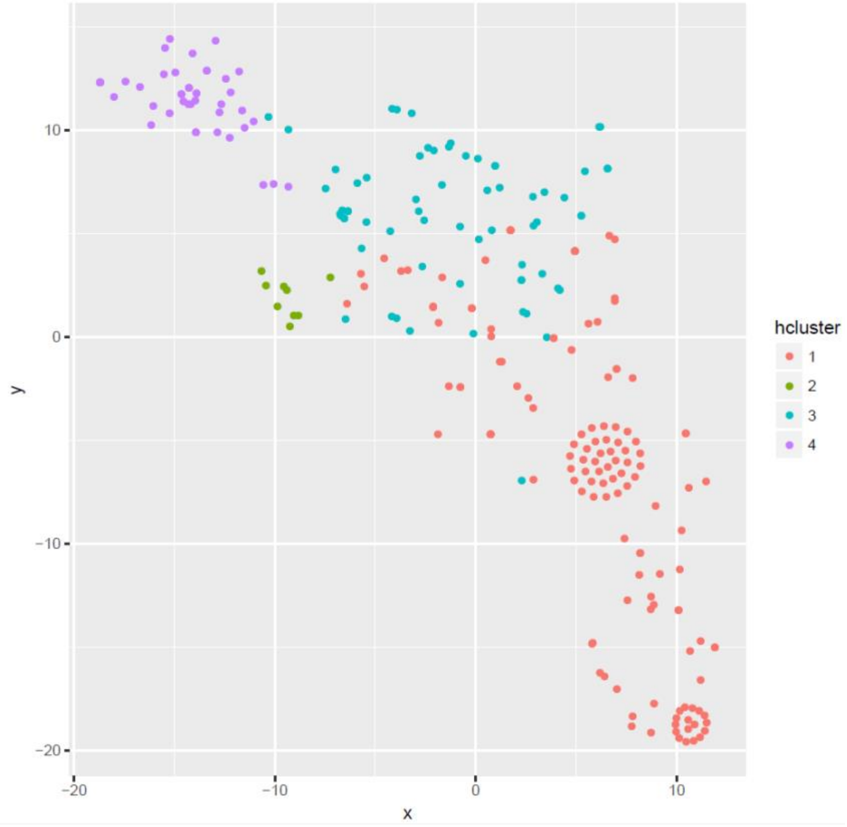

Suppl. Figure 5: Hierarchical clusters (K=4) mapped against Euclidean TSNE projections

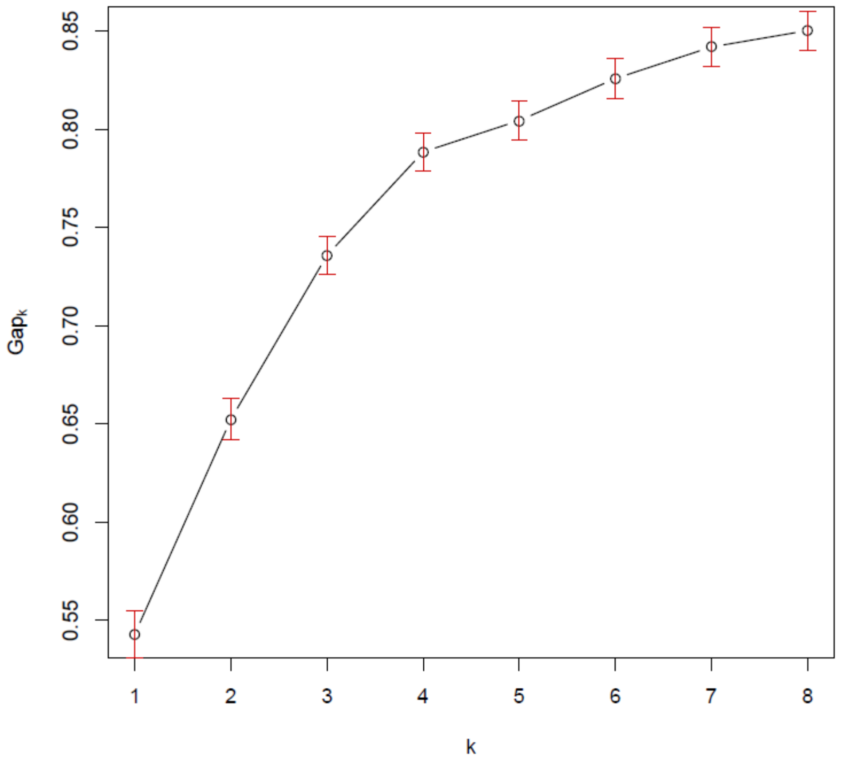

Suppl. Figure 6: Gap statistic computed for kmeans clustering of eHEALS items

References

1. Hastie T, Tibshirani R, Friedman J. The Elements of Statistical Learning: Data Mining, Inference, and Prediction, Second Edition. 2nd ed. New York: Springer-Verlag; 2009.

2. Van Der Maaten L, Hinton G. Visualizing Data using t-SNE. J Mach Learn Res. 2008;9:2579–605

3. van der Maaten L, Hinton G. Visualizing non-metric similarities in multiple maps. Machine Learning. 2012;87(1):33-55. doi: 10.1007/s10994-011-5273-4.
