# Supplementary material for: Electronic Health Literacy Among Magnetic Resonance Imaging and Computed Tomography Medical Imaging Outpatients: Cluster Analysis
Source: J Med Internet Res. 2019 Aug 28;21(8):e13423. doi: 10.2196/13423 (PMC6737886; doi:10.2196/13423)
Supplement: Multimedia Appendix 2 [file jmir_v21i8e13423_app2.pdf]

Multimedia Appendix 2: Unconditional item response probabilities for a 4-class model of electronic health literacy

Suppl. Table 1. Unconditional item response probabilities for a 4-class model of electronic health literacy (column headings in brackets are the names allocated to electronic health literacy classes by the authors; text in italics is used to indicate the eHealth Literacy Scale response option within each item with the highest probability of endorsement for each class).

**eHEALS[a] factor**	**eHEALS item**	**Class 1 (low)**	**Class 2 (moderate)**	**Class 3 (high)**	**Class 4 (very high)**
	Responses (score)	ρ (SE[b])			
**Awareness**	**I know what health resources are available on the internet**				
	Strongly agree (5)	0.00 (0.00)	0.00 (0.00)	0.02 (0.00)	*0.53 (0.14)*
	Agree (4)	0.08 (0.06)	0.41 (0.09)	*0.80 (0.06)*	0.27 (0.14)
	Undecided (3)	0.04 (0.03)	*0.56 (0.09)*	0.12 (0.04)	0.10 (0.05)
	Disagree (2)	*0.72 (0.13)*	0.02 (0.02)	0.06 (0.05)	0.08 (0.12)
	Strongly disagree (1)	0.16 (0.13)	0.02 (0.02)	0.00 (0.00)	0.02 (0.02)
	**I know where to find helpful health resources on the internet**				
	Strongly agree (5)	0.00 (0.00)	0.00 (0.00)	0.02 (0.01)	*0.61 (0.19)*
	Agree (4)	0.08 (0.07)	0.40 (0.10)	*0.91 (0.06)*	0.26 (0.16)
	Undecided (3)	0.04 (0.05)	*0.59 (0.10)*	0.00 (0.00)	0.06 (0.00)
	Disagree (2)	*0.74 (0.18)*	0.01 (0.03)	0.07 (0.05)	0.06 (0.08)
	Strongly disagree (1)	0.15 (0.20)	0.00 (0.02)	0.00 (0.00)	0.02 (0.02)
**Skills**	**I know how to find helpful health resources on the internet**				
	Strongly agree (5)	0.00 (0.00)	0.03 (0.02)	0.00 (0.00)	*0.71 (0.16)*
	Agree (4)	0.08 (0.08)	*0.54 (0.10)*	*0.97 (0.03)*	0.22 (0.11)
	Undecided (3)	0.07 (0.05)	0.44 (0.10)	0.02 (0.02)	0.04 (0.07)
	Disagree (2)	*0.73 (0.16)*	0.00 (0.00)	0.01 (0.01)	0.00 (0.00)
	Strongly disagree (1)	0.11 (0.15)	0.00 (0.00)	0.00 (0.00)	0.04 (0.07)

	**I know how to use the internet to answer my questions about health**				
	Strongly agree (5)	0.02 (0.01)	0.03 (0.05)	0.01 (0.01)	*0.90 (0.08)*
	Agree (4)	0.15 (0.13)	*0.58 (0.10)*	*0.97 (0.03)*	0.08 (0.06)
	Undecided (3)	0.17 (0.07)	0.38 (0.09)	0.00 (0.00)	0.02 (0.04)
	Disagree (2)	*0.48 (0.17)*	0.00 (0.00)	0.02 (0.02)	0.00 (0.00)
	Strongly disagree (1)	0.18 (0.19)	0.02 (0.02)	0.00 (0.00)	0.00 (0.00)
	**I know how to use the health information I find on the internet to help me**				
	Strongly agree (5)	0.00 (0.00)	0.00 (0.00)	0.00 (0.00)	*0.79 (0.10)*
	Agree (4)	0.08 (0.09)	0.47 (0.08)	*1.00 (0.00)*	0.12 (0.09)
	Undecided (3)	0.17 (0.08)	*0.48 (0.07)*	0.00 (0.00)	0.10 (0.10)
	Disagree (2)	*0.57 (0.19)*	0.05 (0.04)	0.00 (0.00)	0.00 (0.00)
	Strongly disagree (1)	0.18 (0.15)	0.00 (0.00)	0.00 (0.00)	0.00 (0.00)
**Evaluate**	**I have the skills I need to evaluate the health resources I find on the internet**				
	Strongly agree (5)	0.00 (0.00)	0.03 (0.04)	0.04 (0.03)	*0.86 (0.10)*
	Agree (4)	0.06 (0.09)	0.24 (0.07)	*0.81 (0.05)*	0.10 (0.07)
	Undecided (3)	0.13 (0.08)	*0.55 (0.09)*	0.09 (0.04)	0.04 (0.05)
	Disagree (2)	*0.57 (0.19)*	0.18 (0.09)	0.05 (0.03)	0.00 (0.00)
	Strongly disagree (1)	0.24 (0.22)	0.00 (0.00)	0.01 (0.02)	0.00 (0.00)
	**I can tell high-quality from low-quality health resources on the internet**				
	Strongly agree (5)	0.02 (0.01)	0.03 (0.02)	0.04 (0.03)	*0.74 (0.10)*
	Agree (4)	0.04 (0.05)	0.14 (0.06)	*0.71 (0.07)*	0.16 (0.07)
	Undecided (3)	0.07 (0.10)	*0.63 (0.09)*	0.15 (0.05)	0.10 (0.09)
	Disagree (2)	*0.69 (0.16)*	0.19 (0.09)	0.10 (0.05)	0.00 (0.00)
	Strongly disagree (1)	0.19 (0.14)	0.01 (0.02)	0.00 (0.00)	0.00 (0.00)
	**I feel confident in using information from the internet to make health decisions**				
	Strongly agree (5)	0.02 (0.04)	0.00 (0.00)	0.00 (0.00)	*0.57 (0.13)*
	Agree (4)	0.06 (0.06)	0.14 (0.06)	*0.68 (0.06)*	0.22 (0.09)
	Undecided (3)	0.13 (0.06)	*0.61 (0.08)*	0.21 (0.05)	0.14 (0.09)

	Disagree (2)	*0.48 (0.12)*	0.21 (0.08)	0.10 (0.04)	0.06 (0.04)
	Strongly disagree (1)	0.31 (0.12)	0.03 (0.03)	0.01 (0.01)	0.02 (0.04)

[a]eHEALS: eHealth Literacy Scale.

[b]SE: standard error.
