# Supplementary material for: Electronic Health Literacy Among Magnetic Resonance Imaging and Computed Tomography Medical Imaging Outpatients: Cluster Analysis
Source: J Med Internet Res. 2019 Aug 28;21(8):e13423. doi: 10.2196/13423 (PMC6737886; doi:10.2196/13423)
Supplement: Multimedia Appendix 3 [file jmir_v21i8e13423_app3.pdf]

Multimedia Appendix 3: Log likelihood difference tests

Suppl. Table 2: Log likelihood difference tests to identify covariates that significantly improved model fit

**Variable**	**Chi-Squared (DF)**	***P*-value**
Age	9.61 (3)	$P = .02$
Gender	4.55 (3)	$P = .21$
Education	508.01 (3)	$P < .001$
Geographic location	2.28 (3)	$P = .52$
Marital status	489.70 (3)	$P < .001$
Information amount preference	526.26 (3)	$P < .001$
Overall health	508.53 (3)	$P < .001$
Internet use frequency	23.95 (3)	$P < .001$
